# Requiring Caution in the Interpretation of Donor‐Derived Cell‐Free DNA Levels During Cancer Treatment in a Kidney Transplant Recipient.: A Case Report

**DOI:** 10.1002/iju5.70180

**Published:** 2026-04-17

**Authors:** Nanaka Katsurayama, Toshihito Hirai, Yu Kijima, Hiroki Ishihara, Hironori Fukuda, Kazuya Omoto, Tomokazu Shimizu, Masashi Inui, Hideki Ishida, Toshio Takagi

**Affiliations:** ^1^ Department of Urology Tokyo Women's Medical University Shinjuku Japan; ^2^ Division of Transplant Management Tokyo Women's Medical University Shinjuku Japan; ^3^ Department of Urology, Yachiyo Medical Center Tokyo Women's Medical University Shinjuku Japan

**Keywords:** avelumab, axitinib, donor‐derived cell‐free DNA, immune checkpoint inhibitor

## Abstract

**Introduction:**

Donor‐derived cfDNA (dd‐cfDNA) indicates allograft rejection when > 1% of total cfDNA, but tumor‐derived cfDNA can interfere in cancer patients.

**Case Presentation:**

A 61‐year‐old male kidney transplant recipient with metastatic renal cell carcinoma (RCC) initiated avelumab and axitinib. Following two cycles of Immno‐oncology (IO) therapy, serum creatinine (Cr) levels increased to 3.1 mg/dL. Although %dd‐cfDNA remained below the conventional 1% threshold, it rose to 0.41%, representing a relative change value (RCV) of +178% from baseline. Two weeks later, Cr increased to 4.5 mg/dL, accompanied by severe hyponatremia, necessitating discontinuation of IO therapy, treated with methylprednisolone and resumed IO therapy; however, Cr remained elevated; he required hemodialysis.

**Conclusion:**

RCV from baseline %dd‐cfDNA may serve as a more reliable indicator of allograft injury, facilitating earlier intervention in transplant recipients undergoing IO therapy.

## Keynote Message

We reported a kidney transplant recipient with metastatic renal cell carcinoma who developed graft dysfunction during IO therapy, despite %dd‐cfDNA remaining below the conventional 1% threshold. However, the relative change value (RCV) of %dd‐cfDNA rose significantly from baseline, preceding allograft failure. This case highlights that %dd‐cfDNA RCV may serve as a sensitive indicator of graft injury in transplant recipients undergoing IO therapy.

**Abbreviations**
Ave: Avelumab
AXI: Axitinib
dd‐cfDNA: Donor‐derived cell‐free DNA
EVR: Everolimus
HD: Hemodialysis
MMF: Mycophenolate Mofetil
MP: Methylprednisolone
RCV: The Relative Change Value
Tac: Tacrolimus

## 1 Introduction

Donor‐derived cell‐free DNA (dd‐cfDNA) is a novel biomarker for detecting allograft rejection [1]. A dd‐cfDNA percentage (%dd‐cfDNA) exceeding 1% of total cell‐free DNA (cfDNA) is commonly used as a threshold to suspect rejection. However, in patients with cancer, the %dd‐cfDNA may be confounded by the presence of cancer‐derived cfDNA, leading to potential misinterpretation. To address this limitation, the RCV, representing the percentage increase between consecutive dd‐cfDNA measurements, has been proposed as an additional indicator for clinical decision‐making [2, 3].

## 2 Case Presentation

**FIGURE 1:**
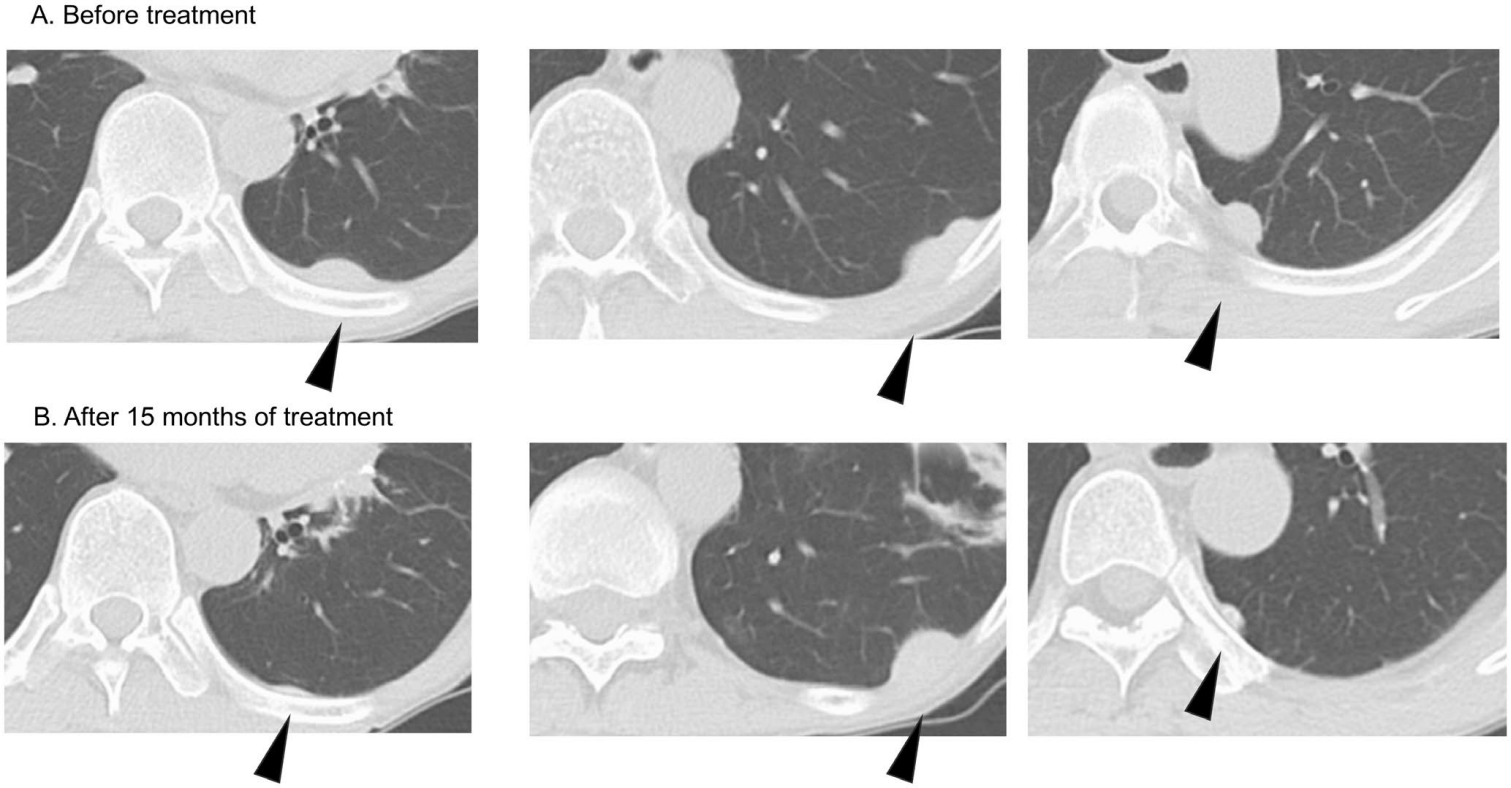
Abdominal computed tomography scan showing metastatic diseases before IO therapy and 12 months after. CT scan at diagnosis shows three pleural metastatic nodules (A). Fifteen months after the IO therapy, all metastatic lesions showed significant size reduction (B).

A 61‐year‐old male underwent living‐donor kidney transplantation. Eleven years later, he developed clear cell RCC, treated surgically. Twenty‐one years post‐transplant, Computed Tomography (CT) showed a left pleural mass raising suspicion of further RCC metastases (Figure 1A). He began immuno‐oncology (IO) therapy with avelumab and axitinib. At baseline, his Cr was 2.4 mg/dL; however, a protocol kidney allograft biopsy performed 15 years after transplantation for the evaluation of proteinuria showed no signs of rejection. During IO therapy, immunosuppressive agents were adjusted as follows: mycophenolate mofetil was discontinued, the tacrolimus dose was reduced from 3 mg/day to 1 mg/day, and methylprednisolone was continued.

**FIGURE 2:**
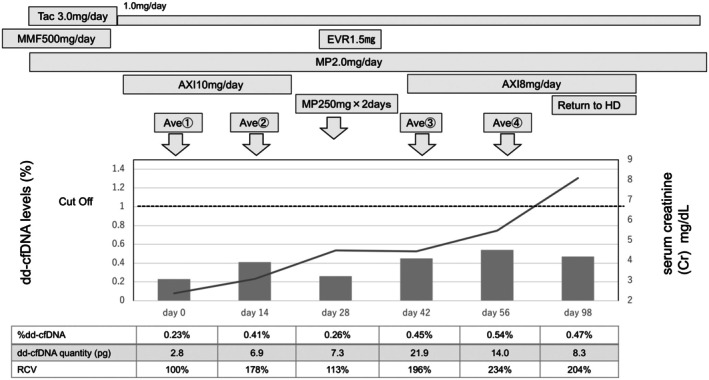
Time course showing serum creatinine level, %dd‐cfDNA, and the relative change value of %dd‐cfDNA with treatment history. Plasma samples for dd‐cfDNA were collected approximately every two weeks during IO therapy. Arrows indicate administration of avelumab (Ave), steroid pulse therapy (MP), and major treatment events including return to hemodialysis. Bar graph: The fraction of donor‐derived cell‐free DNA (dd‐cfDNA, left Y‐axis, %). Dot horizontal line indicates cut‐off level for rejection diagnosis (1%). Line graph; serum creatinine level (right Y‐axis, mg/dL). The relative change value (RCV) was calculated as below: [%dd‐cfDNA]/[%dd‐cfDNA at week 0 (0.23%)] × 100 (%). AXI, Axitinib; dd‐cfDNA, Donor‐derived cell‐free DNA; EVR, Everolimus; HD, Hemodialysis; MMF, Mycophenolate Mofetil; RCV, The Relative Change Value; Tac, Tacrolimus.

The clinical course is shown in Figure 2. After two IO cycles, his Cr rose to 3.1 mg/dL and later to 4.5 mg/dL, with severe hyponatremia. IO therapy was paused, and steroid treatment improved sodium levels, but Cr remained elevated. Given the degree of renal dysfunction and the potential procedural risks, an allograft biopsy was not pursued at that time. Fourteen weeks after initiating IO therapy, Cr reached 8.09 mg/dL, and dialysis was started. Throughout therapy, plasma samples were collected biweekly, and %dd‐cfDNA levels were retrospectively analyzed. Although levels stayed below the 1% rejection threshold, they showed a progressive increase. The RCV from baseline reached +178% after two IO cycles and + 234% at graft failure, while %dd‐cfDNA never exceeded 0.5%.

## 3 Discussion

%dd‐cfDNA has demonstrated potential as a biomarker for the early detection of acute rejection in renal transplantation, with levels exceeding 1% indicative of active rejection. A lower threshold of 0.5% was also suggested to detect subclinical early T cell mediated rejection [4]. However, the variability of serum cfDNA levels, influenced by the recipient's health condition, raises the possibility that the %dd‐cfDNA cut‐off may also be affected by recipient‐specific factors. Then, the absolute quantity of dd‐cfDNA may help detect allograft rejection when %dd‐cfDNA is underestimated due to increased recipient‐derived cfDNA, warranting further investigation [5, 6]. Furthermore, cfDNA levels have been shown to correlate with the presence of metastatic disease [7, 8]. Also, previous studies have suggested during ICI therapy, cfDNA/ctDNA levels may transiently increase due to immune‐mediated tumor cell death, decrease with tumor response, or rise again with disease progression [9, 10]. These findings suggest that in recipients with autologous cancer, particularly metastatic disease, elevated levels of cancer‐derived cfDNA may dilute the dd‐cfDNA, potentially leading to an underestimation of %dd‐cfDNA. These considerations highlight the need for further refinement and contextualization of %dd‐cfDNA thresholds in recipients with malignancies or undergoing myelosuppressive treatments. Our patient had metastatic RCC, suggesting that elevated plasma cancer‐derived cfDNA may have diluted %dd‐cfDNA, thereby reducing its sensitivity for detecting graft rejection. In this study, we retrospectively evaluated RCV because %dd‐cfDNA alone was insufficient to differentiate rejection in the presence of cancer‐derived cfDNA. While the established %dd‐cfDNA cut‐off for rejection is 1%, rejection can still be inferred by calculating the RCV, even when %dd‐cfDNA remains below this threshold. Fluctuations in %dd‐cfDNA of up to 61% are considered within the range of normal biological variation [2]. For example, Laila L et al. reported a case where Cr levels increased to 1.3 mg/dL during pembrolizumab therapy, but %dd‐cfDNA levels remained below 1% and increased by less than 61% from the previous test, making rejection unlikely and allowing continuation of IO therapy [11]. In our case, the patient exhibited a slight increase in serum Cr levels after two cycles of IO therapy, while %dd‐cfDNA was less than 1%, and even below the lower threshold of 0.5%. However, the patient subsequently developed significant renal dysfunction with severe hyponatremia, posing a life‐threatening risk. Notably, the RCV had already risen to 178% two weeks before emergent hospital admission. These findings suggest that an elevated RCV may serve as an early indicator of graft injury, even when %dd‐cfDNA remains below the 1% threshold. Since dd‐cfDNA analysis was retrospectively performed in this study, this information was not available for clinical decision‐making. Given that this is a single retrospective observation, the findings should be considered hypothesis‐generating, and further studies are required to determine whether longitudinal changes in %dd‐cfDNA, including RCV, may have clinical utility in transplant recipients undergoing IO therapy.

This case has limitations, notably the absence of an allograft biopsy due to the patient's unstable condition and preference for cancer treatment over graft preservation.

The exact etiology of graft dysfunction remains uncertain because an allograft biopsy was not performed. Possible causes include acute rejection, ICI‐related interstitial nephritis, drug toxicity, or ATN associated with severe hyponatremia. Therefore, the clinical course may be interpreted as graft dysfunction of uncertain etiology. Although biopsy could have clarified the cause, the risk of irreversible injury was high, and the patient chose to continue IO therapy. This case illustrates the complex decisions needed to balance cancer treatment with graft preservation. In late‐stage post‐transplant dysfunction, biopsy is often difficult; in such cases, %dd‐cfDNA may serve as a useful non‐invasive tool for detecting tissue injury and guiding clinical decisions. Additionally, in this case, %dd‐cfDNA levels were extremely low before cancer treatment, raising concerns that circulating tumor DNA (ctDNA) may have influenced the measurements. We retrospectively examined the total cfDNA concentration extracted from plasma; however, compared to cancer‐free patients, this case did not show a significant increase (data not shown). However, since total cfDNA levels are influenced by multiple pre‐analytical and biological factors, including plasma volume, leukocyte lysis, and systemic inflammation, the lack of elevation in total cfDNA does not contradict the possibility that cancer‐derived cfDNA diluted dd‐cfDNA. ctDNA levels may transiently increase following cancer treatment or decrease due to tumor burden reduction, making the direct interpretation of post‐treatment %dd‐cfDNA levels difficult. When an increase in dd‐cfDNA RCV is observed, all possible factors should be considered in determining the appropriate treatment strategy.

## 4 Conclusion

This case highlights that the observed increase in RCV preceding graft dysfunction suggests that longitudinal changes in %dd‐cfDNA may provide additional information in complex clinical settings. However, further studies are required to clarify the clinical significance of RCV monitoring.

## Funding

This work was supported by Japan Society for the Promotion of Science, JP23K08039.

## Ethics Statement

The study protocol was approved by the institutional ethics review board of Tokyo Women's Medical University (ID: 2021–0115).

## Consent

Written informed consent was obtained from the patient for publication of this case report and the accompanying images.

## Conflicts of Interest

The authors declare no conflicts of interest.

## Data Availability

The data that support the findings of this study are available on request from the corresponding author. The data are not publicly available due to privacy or ethical restrictions.
